# Effective delivery of mycophenolic acid by oxygen nanobubbles for modulating immunosuppression

**DOI:** 10.7150/thno.41850

**Published:** 2020-03-04

**Authors:** Muhammad Saad Khan, Jae-Sung Kim, Jangsun Hwang, Yonghyun Choi, Kyungwoo Lee, Yejin Kwon, Jaehee Jang, Semi Yoon, Chul-Su Yang, Jonghoon Choi

**Affiliations:** 1 School of Integrative Engineering, Chung-Ang University, Seoul 06974, Republic of Korea.; 2 Department of Bionano Technology, Hanyang University, Seoul 04673, Republic of Korea.; 3 Department of Molecular & Life Science, Hanyang University, Ansan 15588, Republic of Korea.

**Keywords:** mycophenolic acid, oxygen nanobubbles, immunosuppression, peripheral blood mononuclear cells, inflammation, autoimmune diseases

## Abstract

Immunosuppressive drugs are crucial for preventing acute graft rejection or autoimmune diseases. They are generally small molecules that require suitable drug carriers for ensuring stability, bioavailability, and longer half-life. Mycophenolic acid (MPA) is an extensively studied immunosuppressive drug. However, it requires suitable carriers for overcoming clinical limitations. Currently, lipid-shelled micro- and nanobubbles are being thoroughly investigated for diagnostic and therapeutic applications, as they possess essential properties, such as injectability, smaller size, gaseous core, high surface area, higher drug payload, and enhanced cellular penetration. Phospholipids are biocompatible and biodegradable molecules, and can be functionalized according to specific requirements.

**Methods**: In this study, we synthesized oxygen nanobubbles (ONBs) and loaded the hydrophobic MPA within the ONBs to generate ONB/MPA. Peripheral blood mononuclear cells (PBMCs) were treated with ONB/MPA to determine the suppression of immune response by measuring cytokine release. *In vivo* murine experiments were performed to evaluate the effectiveness of ONB/MPA in the presence of inflammatory stimulants.

**Results**: Our results suggest that ONBs successfully delivered MPA and reduced the release of cytokines, thereby controlling inflammation and significantly increasing the survival rate of animals.

**Conclusion**: This method can be potentially used for implantation and for treating autoimmune diseases, wherein immunosuppression is desired.

## Introduction

Nanocarriers have been extensively studied for therapeutic applications, particularly for drug delivery 1,2. Nanobubbles consist of a shell composed of biocompatible molecules such as phospholipids, proteins, polymers, and surfactants, encapsulating a core gas such as oxygen, nitric oxide, perfluorocarbons, or air. The shell stabilizes the gas in the core, and as the uncoated gas dissolves rapidly, various gases can be delivered as the core gas in the nanobubbles 3-6. Owing to their size (nanometer range), higher surface area, and higher cellular uptake, these bubbles are also suitable for drug delivery as they can be used to incorporate the drugs either in the shell (via hydrophobic interactions), or inside the core 6-12. Phospholipid shells are favorable due to their resemblance to cell membrane structure, stability at nanosize, ease in synthesis and ability to load both hydrophilic and hydrophobic drugs 8,13.

Immunosuppressive drugs suffer from issues like low solubility, rapid clearance, structural instability, requirement of high dosage and therefore they need biocompatible drug carriers to increase bioavailability, reduce required dosage and improve the effectiveness for superior immunosuppression 14-20. Mycophenolic acid (MPA) is a hydrophobic immunosuppressive drug with an antiproliferative mechanism of action with respect to T and B lymphocytes. In addition to its immunosuppressive effects, MPA has a broad spectrum of antifungal, antibacterial, antiviral, and antitumor effects 21-31. MPA is rapidly metabolized in the liver and quickly excreted from the body and therefore it has low bioavailability 32. Studies have shown increased incidence of secondary infections after MPA administration 31, 33-35. A suitable drug delivery system may therefore enhance bioavailability at a lower dosage of MPA, thereby minimizing the adverse effects. In addition, an optimal immunosuppressive drug delivery system that can control hyperinflammation without compromising the pathogen clearance capability of the host is required 25.

Hypoxia inducible factor 1alpha (HIF-1α) has been associated with inflammation in various studies. Hypoxia and inflammation have interdependent and bidirectional association and they influence one another. Downregulation and inhibition of HIF-1α has been reported to have a key role in reducing inflammation in a lung injury model 36-48. In the presence of oxygen, procollagen prolyl hydroxylase domain (PHD)-containing enzymes hydroxylate HIF-1α, resulting in its downregulation 39. In our previous study, we synthesized oxygen nanobubbles (ONBs) and characterized oxygen delivery via ONBs, leading to HIF-1α downregulation and reversal of hypoxia 49. In this study, we aimed to downregulate HIF-1α, which might prove to be beneficial for suppressing the immune response 42,50.

As mentioned before, MPA is a hydrophobic drug which can be administered with a suitable drug carrier. The role of oxygen nanobubbles in the downregulation of HIF-1α has also been significantly researched, and HIF-1α upregulation has been observed during inflammation. Therefore, we hypothesize that ONBs will effectively encapsulate MPA and improve its efficacy by increasing its bioavailability and biodistribution, and by providing oxygen to downregulate HIF-1α. To the best of our knowledge, MPA delivery via ONBs is a novel idea that has not been previously reported. In this study, we encapsulated the hydrophobic immunosuppressive drug MPA in phospholipid-shelled ONBs (ONB/MPA) and evaluated its immunosuppressive and anti-inflammatory effects. We also investigated the role of oxygen in cytokine release from peripheral blood mononuclear cells (PBMCs) under normal and hypoxic conditions. Lipopolysaccharide (LPS) induces inflammation and increases pro-inflammatory cytokine release with strong inflammatory effects, while cecum ligation and puncture (CLP) is used as a standard model of human sepsis 25,51. We investigated the relationship between HIF-1α and the CLP-induced sepsis model to analyze the effect of ONB/MPA on HIF-1α downregulation. Additionally, we investigated the biodistribution of Cy5.5-labeled ONBs (ONB/Cy5.5) through an *in vivo* imaging system and evaluated fluorescence in spleen, liver, lung, and kidney tissues. We also evaluated the adaptive immune cells present in these tissues. We performed immunohistopathological analysis of lung, liver, spleen, kidney, and large intestine tissues to evaluate the effect of our proposed ONB/MPA. We believe that this study will play a pivotal role in devising an immunosuppressive drug delivery system for controlling excessive inflammation and providing sufficient immunosuppression during implantation and in autoimmune diseases, while avoiding compromising the ability of the host to clear the pathogens.

## Results & Discussion

In this study, we investigated the ability of ONBs to deliver immunosuppressive MPA and evaluated the role of ONB/MPA in reducing inflammation.

### Characterization of ONBs and ONB/MPA

**Figure 1:**
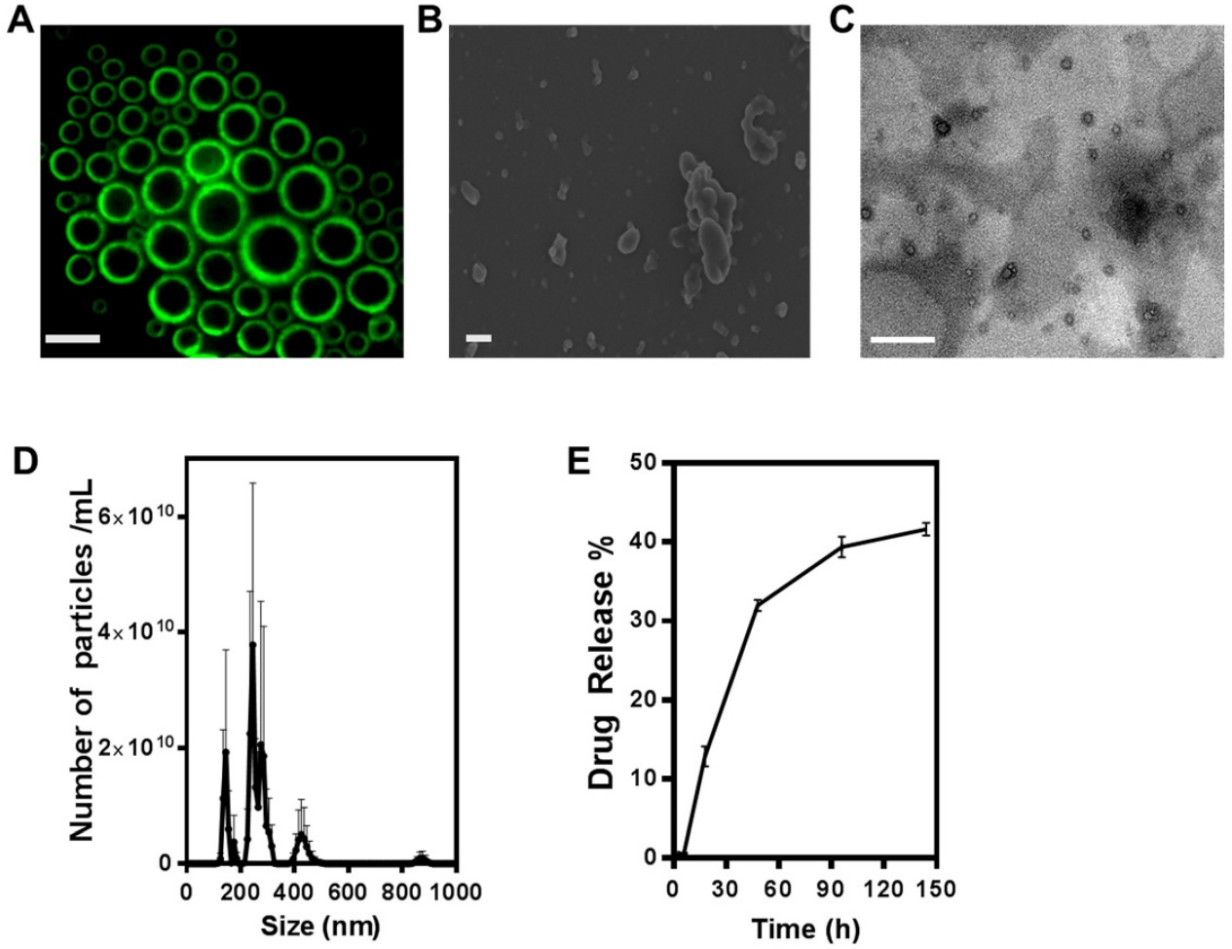
Characterization of oxygen nanobubbles (ONB)/mycophenolic acid (MPA). (A) Fluorescence image of micro-sized oxygen bubbles labelled with FITC (scale bars = 10 µm). (B) SEM image of ONB, scale bar = 1 µm, (C) TEM image of ONB, scale bar = 100 nm. (D) Size distribution and the number of ONBs measured using the nanoparticle tracking analyzer (NTA) system. (E) The drug release profile of ONB/MPA. Absorbance was measured at 430 nm.

Figure 1A shows the result of constructing fluorescent microbubbles and clearly indicates a shell/core composition. For this purpose, micron size bubbles were collected and observed under fluorescence microscope. Figure 1B shows the SEM image of ONB. For this purpose, ONB sample was rapidly frozen using liquid nitrogen, which resulted in some aggregation of ONB, showing irregular shapes but many individual ONB are clearly visible in the image. TEM image of nanobubbles are shown in Figure 1C, indicating the shell/core composition of ONB. Size distribution was measured using a nanoparticle tracking analyzer (NTA), as shown in Figure 1D. The mean size was 261 ± 41 nm, with a significant peak at 245 nm. The sonication method was used to synthesize ONB and ONB/MPA samples. Being a stochastic method, sonication generates both micro- and nanosize bubbles, for which detailed characterization and oxygen delivery has been reported in our previous studies 37,52,53. This size (<500 nm) is favorable for endocytosis, enhanced permeability, and retention effect 3,52. The particle concentration measured through NTA was 4.4 ± 0.78 x 10^11^ nanobubbles/mL. The drug release profile of ONB/MPA is shown in Figure 1E. An approximate 40% release was observed after 144 h, indicating a slow but consistent diffusion. These observations indicated the stability of ONB/MPA and the extended bioavailability of the drug. Higher bioavailability enhances the effectiveness of the drug, as observed in case of both *in vitro* and *in vivo* assays. We did not use ultrasound for disrupting the bubbles, thereby eliminating the issues associated with high-intensity ultrasound. Our results indicate that drugs can be released from ONBs via diffusion. Theoretically, the release of the MPA encapsulated inside the shell requires disruption of the shell of the bubble. After oxygen diffuses out of the shell, the bubble shrinks to a point where the external Laplace pressure breaks the bubble, resulting in release of the drug 8,53,54. This process is slower than destruction of bubbles using high-intensity ultrasound. Nanobubbles are also taken up by the cells via endocytosis and are metabolically degraded to release the encapsulated drug 55.

Figure S1 shows the biological properties of ONB/MPA *in vivo*. Figure S1A shows the absorption spectra of MPA, while S1B shows the standard curve obtained from the absorption spectra. Figure S1C shows that ONBs and lipids were not significantly cytotoxic to PBMCs after treatment with different concentrations (up to 50 µL/mL (1:20 (v/v), ONB media) of ONBs and lipid constituents. Figure S1D shows the cell viability after treatment with different concentrations of MPA, whereas Figure S1E shows the cell viability after treatment with ONBs, MPA, phytohemagglutinin (PHA), ONB+MPA, and ONB+MPA+PHA. The viability of all samples was similar, with no significant differences in cytotoxicity.

### Cytokine profiling of ONB constituents

**Figure 2:**
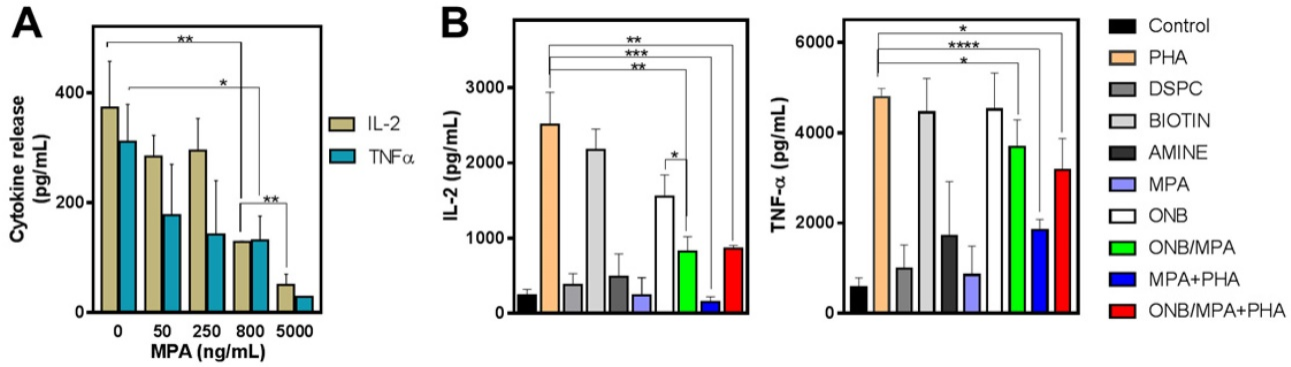
Cytokine profile after various treatments. (A) IL-2 and TNF-α release after treatment of peripheral blood mononuclear cells (PBMCs) with various concentrations of mycophenolic acid (MPA). (B) IL-2 and TNF-α profile after treatment of PBMCs with oxygen nanobubbles (ONBs), MPA, phorbol-12-myristate-13-acetate (PHA), ONB/MPA, DSPC, DSPE-PEG-Amine (shown as Amine), DSPE-PEG-2000-Biotin (shown as Biotin), and ONB/MPA+PHA (PBMCs: 5 × 10^5^ cells/mL; PHA: 5 µg/mL; MPA: 500 ng/mL; ONBs: 20 µL/mL). *p < 0.05, **p < 0.01, ***p < 0.001,****p < 0.0001.

To study the delivery of MPA via ONB/MPA, we designed *in vitro* experiments for assessing cytokine release from PBMCs. First, we investigated the roles of ONBs, their constituents (DSPC, DSPE-PEG-Amine, and DSPE-PEG-Biotin), and ONB/MPA. Figure 2 shows the results of cytokine profiling after these treatments. In the absence of any external stimulation, MPA reduced the release of IL-2 and TNF-α from PBMCs in a dose-dependent manner (Figure 2A). We performed an enzyme-linked immunosorbent assay (ELISA) to check the immune response to the constituents of ONBs (Figure 2B). PHA was used as a positive control and as a stimulant of IL-6 and TNF-ɑ. PHA stimulated the release of 2,500 ± 433 pg/mL IL-2, and the PHA constituent DSPE-PEG-2000-Biotin stimulated the release of 2,172 ± 277 pg/mL IL-2. There was no significant difference between the positive control PHA and DSPE-PEG-2000-Biotin (Figure 2B). Compared to the untreated control, other constituents of ONBs such as DSPC and DSPE-PEG-Amine did not show any significant immune response. ONBs were composed of DSPC, DSPE-PEG-Amine, and DSPE-PEG-Biotin at a molar ratio of 85:8:7. They elicited the release of 1,550 ± 290 pg/mL IL-2, whereas ONB/MPA significantly reduced the release of IL-2 to 817 ± 200 pg/mL. This indicated that ONB/MPA reduced the release of IL-2 by 40% compared to ONBs. This can be attributed to the successful delivery of MPA via ONBs. ONB/MPA+PHA also exhibited a similar trend, with no significant difference with respect to ONB/MPA treatment in the amount of IL-2 released. A similar trend was observed for TNF-α release. PHA and DSPE-PEG-Biotin showed a similar TNF-α release (4,780 ± 200 pg/mL and 4,450 ± 757 pg/mL, respectively), with no significant difference between the two. ONBs demonstrated a similar TNF-α release of 4,513 ± 808 pg/mL. ONB/MPA+PHA released 3170 ± 710 pg/mL TNF-α, and ONB/MPA reduced the release of TNF-α to 3,680 ± 608 pg/mL. We therefore concluded that DSPE-PEG-2000-Biotin is responsible for the increase in immune stimulation. However, ONB/MPA successfully delivered MPA, as indicated by its usage for reducing the immune response.

To investigate further, we synthesized ONBs without DSPE-PEG-Biotin, using 85:15 DSPC and DSPE-PEG-Amine, to assess suppression of the immune response. Figures S2A and S2B show the IL-2 and TNF-α responses, respectively. Compared to the untreated control, 10% (v/v) ONBs did not significantly increase IL-2 and TNF-α levels. This validates our previous assumption that immune stimulation was due to the presence of DSPE-PEG-2000-Biotin. Phorbol-12-myristate-13-acetate (PMA) is a stimulant for IL-2 and TNF-α production, and served as a positive control in this experiment. Compared to PMA alone, ONB/MPA and ONB/MPA/PMA significantly reduced IL-2 release by more than 400 pg/mL. However, IL-2 release did not vary significantly between ONB/MPA and ONB/MPA/PMA treatments. The causes of immune stimulation due to DSPE-PEG-2000-Biotin are unknown and beyond the scope of this study. Based on the results of the experiments shown in Figures 2 and S2, we changed the composition of our ONB/MPA from DSPC: DSPE-PEG-2000-Amine: DSPE-PEG-2000-Biotin (85:9:7) to DSPC: DSPE-PEG-Amine (85:15). We then performed further experiments and *in vivo* studies using this composition.

### Cytokine release under hypoxic and normal conditions

**Figure 3:**
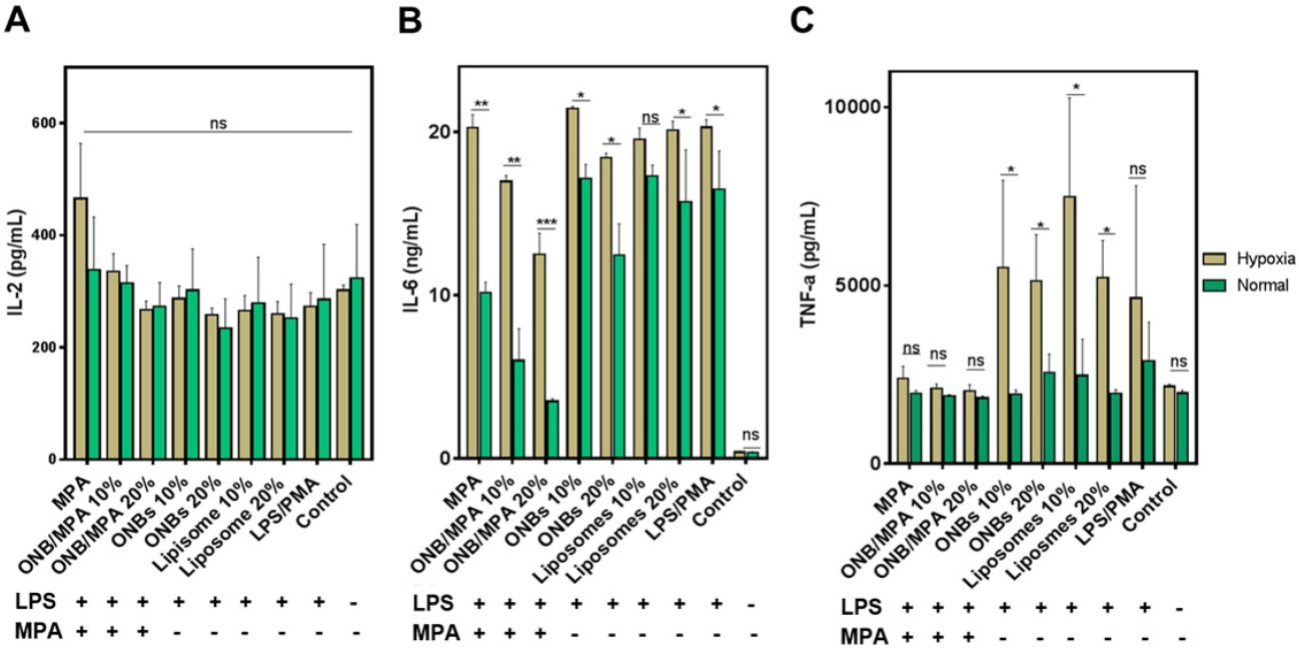
Immunosuppression test of oxygen nanobubbles (ONBs)/mycophenolic acid (MPA) after lipopolysaccharide (LPS) stimulation under hypoxic and normal conditions. (A) IL-2, (B) IL-6, and (C) TNF-α profiling of LPS-stimulated peripheral blood mononuclear cells (PBMCs) under normal and hypoxic conditions (ONB/MPA, total MPA = 600 µg/mL, LPS = 1 µg/mL, phorbol-12-myristate-13-acetate (PMA) = 5 µg/mL. Samples were treated with LPS/PMA prior to hypoxia induction). ns indicates no significance. *p < 0.05, **p < 0.01, ***p < 0.001.

To investigate the anti-inflammatory role of oxygen in immunosuppression, we created normal and mild hypoxic conditions (5% O_2_) for PBMCs and evaluated the release of IL-2, IL-6, and TNF-ɑ. Figure 3 shows the immune response of LPS-stimulated ONB/MPA under hypoxic and normal conditions. Figure 3A indicates that LPS/PMA stimulation did not significantly affect IL-2 release under hypoxic or normal conditions after 48 h of incubation. Similar trends were observed for all treatment conditions after 48 h of incubation. This is consistent with certain previous studies, which indicate that LPS alone does not significantly stimulate IL-2 release in human-derived immune cells 56,57. However, as shown in Figures 2 and S2, PHA and PMA were able to stimulate IL-2, and the ONB/MPA-treated group showed a reduction in IL-2 release. For the results shown in Figure 3, we aimed to coordinate these with planned *in vivo* studies. We therefore opted to perform LPS stimulation. Figure 3B shows IL-6 release under various conditions, and LPS stimulation was performed in all groups save for the control. The liposome and ONB compositions were identical, the only difference being oxygenation occurring for ONBs. Hypoxia increased cytokine release in all cases of LPS stimulation, which may be related to HIF-1α expression and stabilization. LPS/PMA stimulated IL-6 release at 20.28 ± 0.46 ng/mL under hypoxic conditions and 16.48 ± 2.03 ng/mL under normal conditions. When treated with free MPA, the amount of secreted IL-6 was 20.26 ± 0.81 ng/mL under hypoxic conditions and 10.16 ± 0.54 ng/mL under normal conditions. This indicated that after LPS stimulation, free MPA was unable to suppress the immune response under hypoxic conditions. However, under normal conditions, free MPA reduced IL-6 release by 38% compared to LPS/PMA-induced IL-6 release. ONB/MPA (10% and 20% v/v) was able to significantly reduce IL-6 release under both hypoxic and normal conditions. The 10% (v/v) ONB/MPA group showed 16.97 ± 0.36 ng/mL IL-6 release under hypoxic conditions and 6.01 ± 1.68 ng/mL under normal conditions. The 20% (v/v) ONB/MPA group showed 12.5 ± 1.3 ng/mL IL-6 release in hypoxic conditions and 3.5 ± 0.14 ng/mL in normal conditions. This may be due to the successful delivery of MPA via ONBs under all conditions. As the amount of MPA used was similar for both free MPA and ONB/MPA, we can observe the role of oxygen with respect to reducing cytokine release by ONB/MPA 10% (v/v) and ONB/MPA 20% (v/v). This suggested that ONB/MPA was successful in controlling LPS-stimulated inflammation under both normal and hypoxic conditions. When only ONBs were used, higher levels of ONBs (20% v/v) reduced cytokine levels to 18.42 ± 0.21 ng/mL under hypoxic conditions and to 12.44 ± 1.68 ng/mL under normal conditions. Liposomes 10% (v/v) showed an IL-6 release of 19.55 ± 0.71 ng/mL in hypoxic conditions and 17.3 ± 0.59 ng/mL in normal conditions. Liposome 20% (v/v) showed an IL-6 release of 20.1 ± 0.57 ng/mL in hypoxic conditions and 15.7 ± 2.75 ng/mL in normal conditions. The ONB and liposome groups were compared to evaluate the role of oxygen in cytokine release after LPS stimulation. Comparison of these results indicated that oxygen is likely to be involved in HIF-1α degradation and hypoxia reversal, thereby resulting in reduced cytokine release.

Figure 3C shows the TNF-α release profile after various treatments. Free MPA, 10% ONB/MPA, and 20% ONB/MPA reduced TNF-α secretion under both normal and hypoxic conditions. Under hypoxic conditions, TNF-α levels were higher after treatment with 10% (v/v) or 20% (v/v) ONBs and liposomes than after MPA treatment. As hypoxia is associated with infections, it is noteworthy that ONB/MPA was able to suppress LPS/PMA-stimulated TNF-α release, particularly under hypoxic conditions. Taken together, these results indicate that cytokine release was generally higher under hypoxic conditions, and that ONB/MPA was able to successfully deliver MPA. Furthermore, cytokine reduction occurred due to the combined effect of oxygen and successful drug delivery in human-derived PBMCs, particularly under hypoxic conditions. As hypoxia is related to HIF-1ɑ stabilization and upregulation, it is likely that increased oxygen supply helps to reduce cytokine release.

### *In vivo* models of LPS and CLP

**Figure 4:**
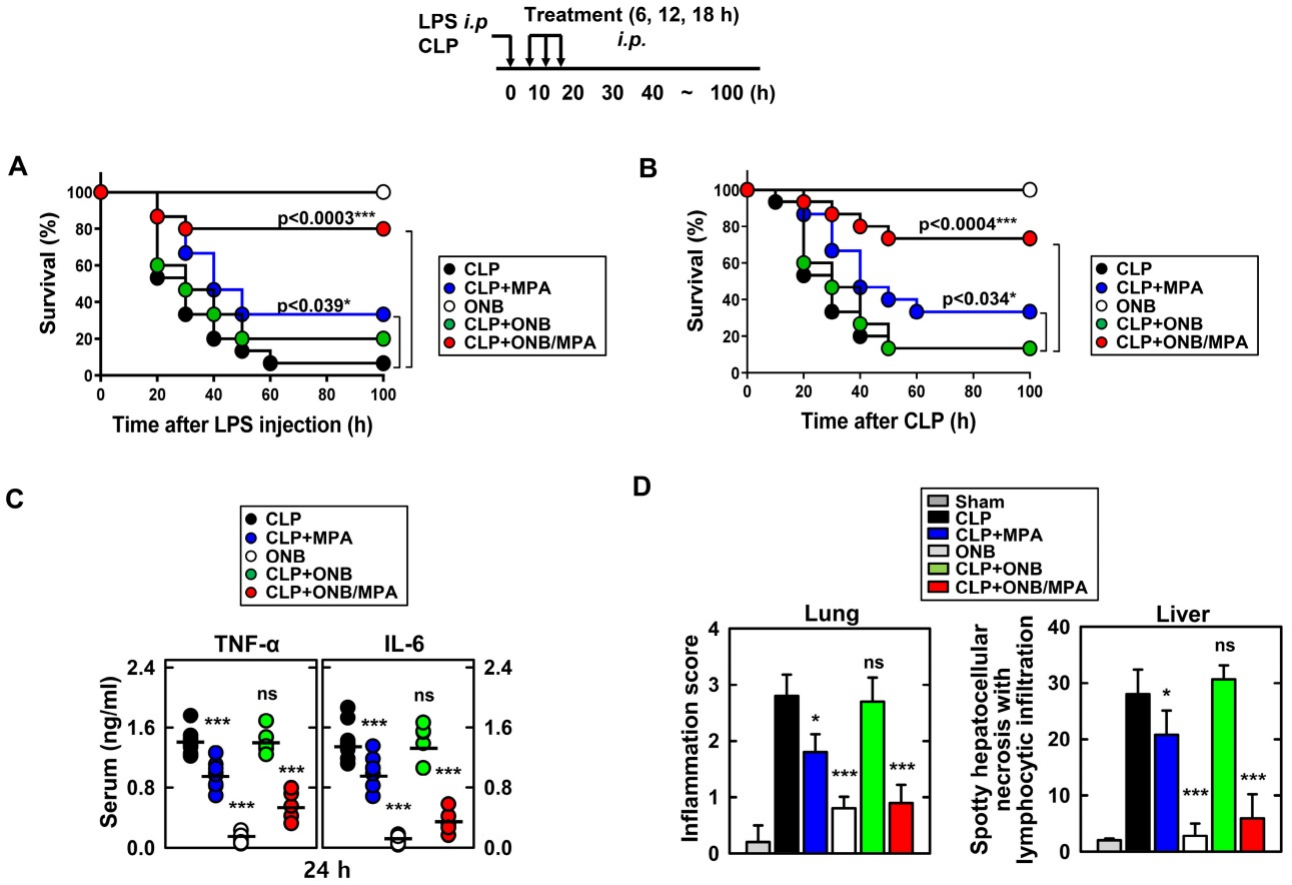
*In vivo* cytokine profiling and immunosuppression. Schematic of the lipopolysaccharide (LPS)/cecum ligation and puncture (CLP) model treated with mycophenolic acid (MPA), oxygen nanobubbles (ONBs), or ONB/MPA (upper). (A) Animal survival after LPS (30 mg/kg, administered intraperitoneally)-induced sepsis (n=15 mice per group). (B) Animal survival after CLP procedure (n=15 mice per group). The data are representative of two independent experiments with similar results. (C) Serum cytokine levels (TNF-α and IL-6) and (D) inflammation score in the lung and liver, obtained by hematoxylin and eosin staining. These were determined at 30 h in CLP mice treated with MPA, ONBs, or ONB/MPA (n=15 mice per group). Significant differences (*p < 0.01; ***p < 0.001) compared with CLP only. Gray bar indicates sham.

Figure 4 shows the results of *in vivo* experiments. Figures 4A and 4B indicate the survival percentages of the different treatment groups in LPS and CLP-induced sepsis models, respectively. Figure 4A indicates that the only ONB treatment was non-toxic to the animals and led to 100% survival. Infection due to LPS stimulation reduced the survival rate to 6.67%, indicating that hyperinflammation caused animal death. Animals treated with LPS+MPA exhibited 33.3% survival, whereas those treated with LPS+ONB/MPA exhibited significantly higher survival (80%). LPS+ON-treated mice also exhibited a survival rate of 20%, which was indicative of the moderate role of oxygen in controlling inflammation. Similar results were observed in the case of the CLP-induced sepsis model (Figure 4B), wherein the survival percentage of CLP+ONB/MPA-treated animals was significantly higher (73.3%) than that of CLP- (13.3%) and CLP+MPA (33.3%)-treated animals. These results along with a 100% survival rate in the ONB group also indicated that ONBs are non-toxic to the animals. Furthermore, ONB/MPA was able to control inflammation, which significantly improved animal survival. Importantly, although hyperinflammation is not desired, the immune response should be adequate to mitigate the risks of secondary infections. The cytokine release profiles (TNF-α and IL-6) were therefore evaluated in the CLP-induced sepsis model, as shown in Figure 4C. A significant reduction in cytokine release was observed after CLP+ONB/MPA treatment compared to that observed after CLP only and CLP+MPA treatment. IL-6 release decreased slightly when animals were treated with CLP+ONB. The group treated with ONB alone did not exhibit TNF-α and IL-6 release.

Figure 4D shows the inflammation score in lung and liver tissue. ONB/MPA-treated animals exhibited inflammation in the mild category compared to animals treated with CLP+MPA and CLP that exhibited moderate and severe inflammation, respectively. The lung and liver inflammation score were in the mild category in the ONB/MPA group, while the CLP control exhibited severe inflammation. These results suggested that ONBs were not involved in activating the immune response, and the immune response was instead reduced adequately when MPA was encapsulated inside ONBs. We also determined that in both LPS and CLP-induced sepsis models, ONB/MPA was able to control inflammation while improving the survival rate and reducing cytokine release.

**Figure 5:**
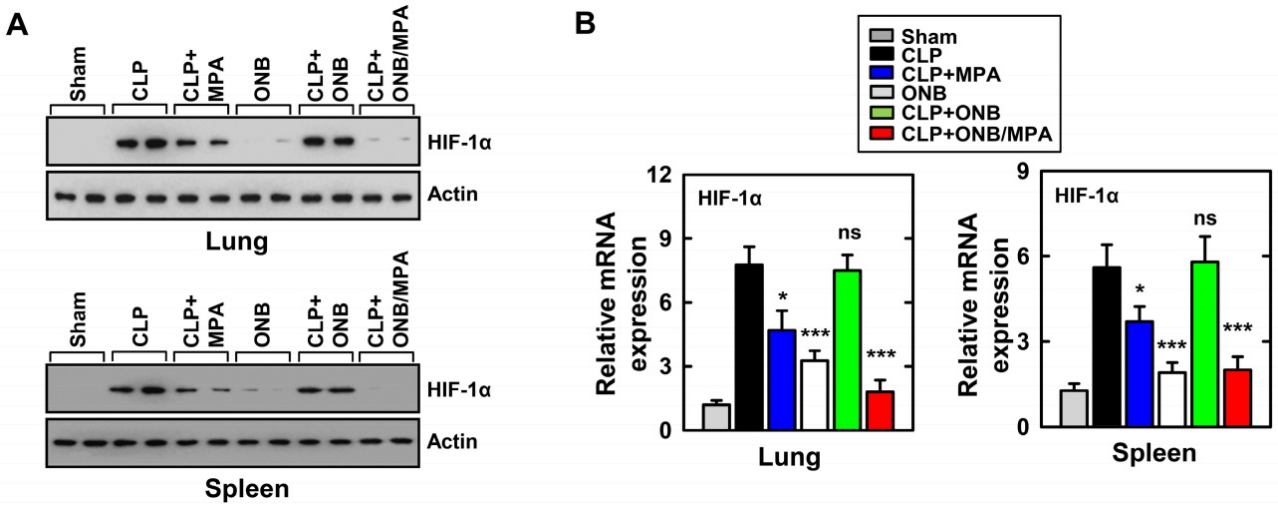
Evaluation of HIF-1α expression in the *in vivo* model. (A) Results of western blotting for αHIF-1α in the lungs and spleen. Loading controls were used for immunoblotting with α-actin. The data are representative of three independent experiments with similar results. (B) Results of real-time polymerase chain reaction using lung and spleen samples were determined at 30 h in cecum ligation and puncture (CLP) mice treated with mycophenolic acid (MPA), oxygen nanobubbles (ONBs), or ONB/MPA (n=15 mice per group). Significant differences (*p < 0.01; ***p < 0.001) compared with CLP only.

As mentioned before, cytokine release was high during *in vitro* experiments under hypoxic conditions. HIF-1α stabilization is a well-known biomarker of hypoxia, and previous studies have demonstrated HIF-1α activation and stabilization during inflammation 37,39,41,45,47. To study the role of HIF-1α expression during inflammation and immunosuppression in our *in vivo* model, western blotting and real-time PCR were used for evaluating HIF-1α protein and mRNA levels, respectively, in the lungs and spleen. Figure 5A shows the results of western blotting. CLP, CLP+MPA, and CLP+ONB treatment stabilized HIF-1α, whereas the CLP+ONB/MPA-treated group did not express HIF-1α either in the lungs or spleen. Figure 5B shows the relative mRNA expression of HIF-1α in the lungs and spleen. ONB/MPA was able to significantly downregulate HIF-1α mRNA expression in the CLP-induced sepsis model. We therefore concluded that HIF-1α expression is high during inflammation, which was downregulated by ONB/MPA. Previous studies have indicated that oxygen supplied via nanobubbles can downregulate HIF-1α, which has a crucial role in inflammation as it stimulates the innate and adaptive immune response 47. We concluded that ONB/MPA has a synergetic effect on controlling infection, suppressing the immune system, and increasing the survival rate.

Figure S4 shows the histological analysis of the liver, lung, and spleen tissues from different treatment groups. Hematoxylin and eosin (H&E) staining of several types of tissue from CLP-treated mice revealed severe pulmonary inflammation with alveolar wall thickening, and necrosis of hepatocytes (increased eosinophilia of cytoplasm and pyknosis) and splenocytes (karyorrhexis). Conversely, ONB/MPA treatment significantly reduced these alterations.

Semiquantitative scoring of these histological parameters (the number and distribution of inflammatory cells within the tissues, as well as non-inflammatory changes such as evidence of bronchiolar epithelial injury and repair) demonstrated that the severity of sepsis in ONB/MPA-treated CLP mice was significantly lower than that in CLP mice (Figure 4D).

**Figure 6:**
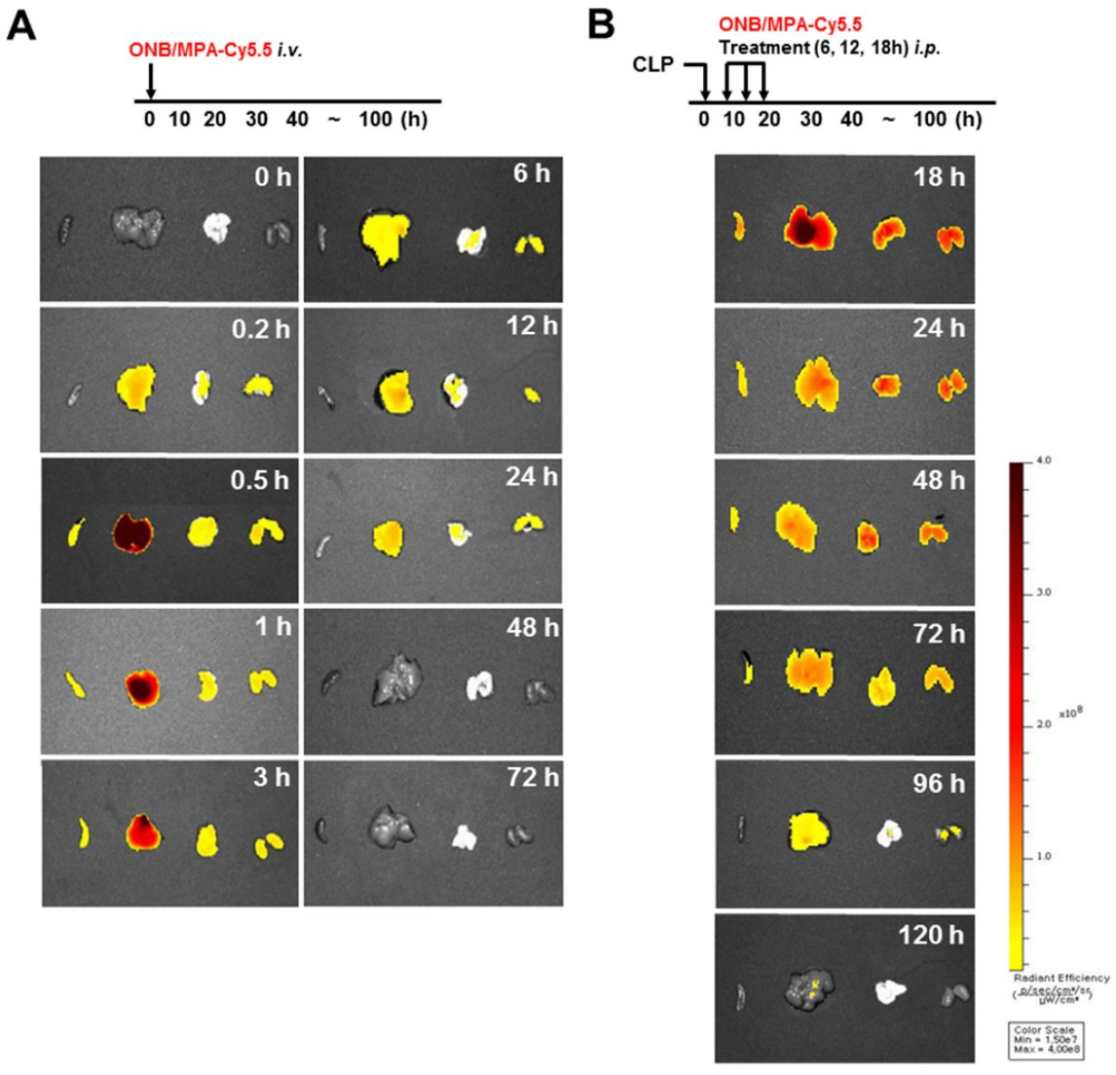
*** In vivo* imaging using IVIS spectrum-chromatography (CT) system.** (A) Pharmacokinetics and biodistribution observed in spleen, liver, lung and kidney tissues after oxygen nanobubble (ONB)/Cy5.5 injection in the tail vain of mice. (B) Intraperitoneal administration of ONB/Cy5.5 was carried out at intervals of 6, 12, and 18 h in a cecum ligation and puncture (CLP)-induced sepsis model. Pharmacokinetics and biodistribution were observed in spleen, liver, lung, and kidney tissues.

Figure 6 shows the biodistribution and pharmacokinetics of nanobubbles in the bodies of mice after infection with fluorescent ONBs (ONB/Cy5.5). Figure 6A shows the results of intravenous administration of ONB/Cy5.5 in the tail vein of mice. After 10 min, ONBs were observed in the liver, lung, and kidney tissues. From 30 min to 3 h, fluorescence indicated high amounts of the sample in the liver. Sample was also present in spleen, lung, and kidney tissues. The sample was visible in the body from 6 to 24 h, after which it appeared to be eliminated from the body. Figure 6B shows the result of intraperitoneal injections of ONB/Cy5.5 at intervals of 6, 12, and 18 h. ONBs were retained in the body for 72 h. They were at their highest concentration in the liver at 18 and 24 h, and were present in comparatively high concentrations in lung and kidney tissues at 48 h. It can therefore be concluded that ONBs were circulating in the body for a significant amount of time and are not rapidly biodegradable. This has significant implications for MPA drug delivery. MMF, a prodrug of MPA, is generally administered twice a day due to rapid metabolism and release from the body 58,59. Therefore, sustained presence in the body and increased bioavailability would reduce the required dosage of MPA. This would in turn reduce the side effects and enhance the effectiveness of the drug.

**Figure 7:**
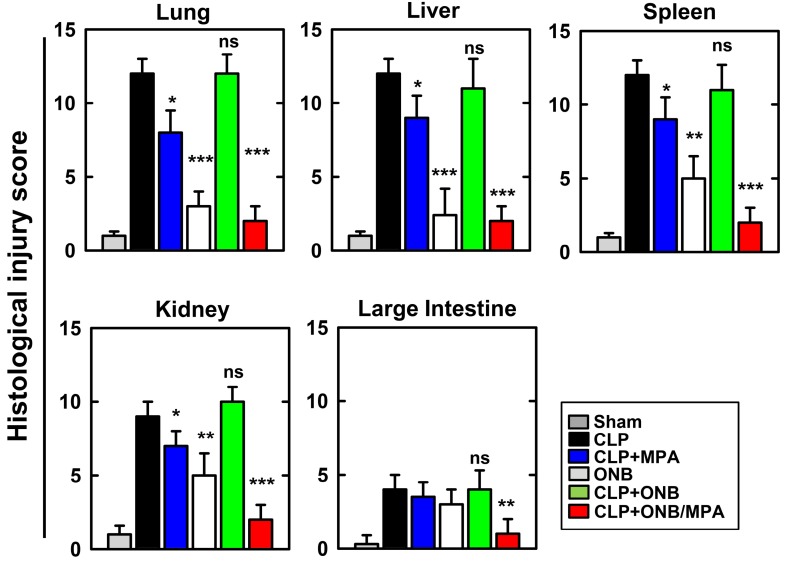
** Histological injury score (HIS) in lung, liver, spleen, kidney, and large intestine tissue.** Histological scores corresponding to the severity of the inflammation (range 0-15) were ascribed to each specimen. Corresponding images are available in Supplementary Figure 4S.

Figure 7 shows the histological injury score (HIS) on a scale of 0-15. Severe CLP-induced inflammation was observed in lung, liver, spleen, and kidney tissues, whereas mild inflammation occurred in the large intestine. These results are in agreement with those of previously published studies. CLP+MPA treatment led to moderate to severe inflammation in the lung, liver, spleen, and kidney tissues. However, ONB/MPA treatment showed only mild inflammation. In the large intestine, CLP and CLP+MPA exhibited mild inflammation, and ONB/MPA was able to alleviate inflammation. These results suggest that ONB/MPA reduced inflammation significantly after CLP induction. A previous study revealed that when the prodrug MMF was introduced after 2 h of CLP induction, it was no longer effective 60. This is because early therapy is crucial for sepsis treatment. In our study, we treated animals after 6 h of CLP induction, and ONB/MPA treatment was still able to control inflammation. Treatment also improved animal survival rate, demonstrating that ONB/MPA is an effective drug carrier. As sepsis is associated with high inflammation, multiple organ injuries, and mortality, reduction in HIS explains the higher animal survival rate shown in Figure 4. Sepsis models share some pathophysiological reactions with organ rejection, and thus the results of ONB/MPA suppression could be extended to prevent organ rejection models 25.

**Figure 8:**
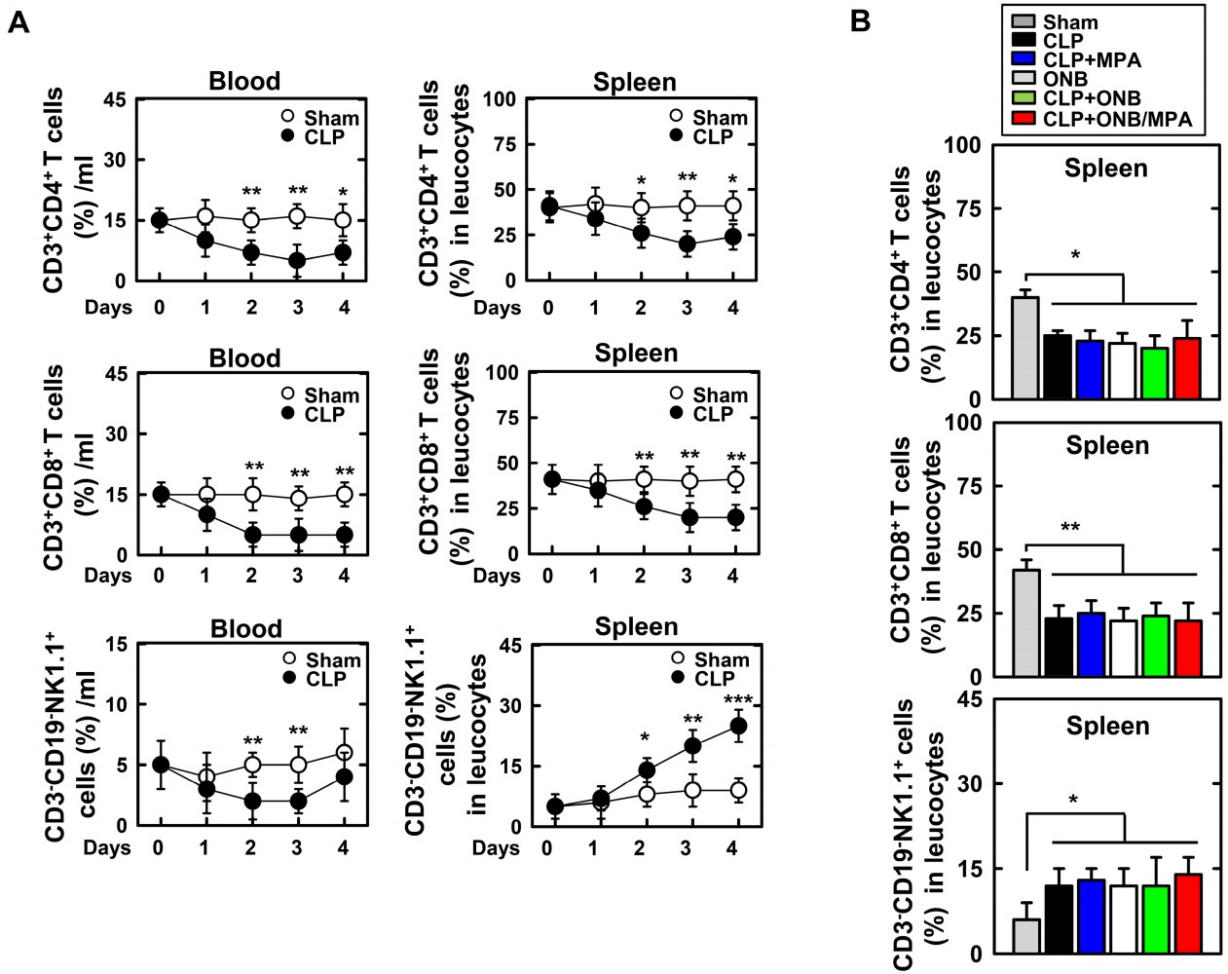
** Fluorescence-activated cell sorting (FACS) analysis of adaptive immune-cells in blood and spleen. A.** The percentage of CD3+CD4+ T cells, CD3+CD8+ T cells, and CD3-CD19-NK1.1+ T cells found in the blood or spleen using FACS analysis in the background of cecum ligation and puncture (CLP)-induced sepsis. **B.** Mice were euthanized after 30 h of CLP induction to evaluate CD3+CD4+ T cells, CD3+CD8+ T cells, and CD3-CD19-NK1.1+ T cells in the spleen.

Experiments were designed to evaluate adaptive immune cells in the blood and spleen (Figure 8). Figure 8A shows the percentage of CD3^+^CD4^+^ T cells, CD3^+^CD8^+^ T cells, and CD3^-^CD19^-^NK1.1^+^ T cells in the blood or spleen using FACS analysis in the background of CLP-induced sepsis. As shown in Figure 8A, CLP induction rapidly decreased the T cell lineage population (1-2 days). However, the population recovered after 4 days. Interestingly, natural killer (NK) cell counts were only increased in the spleen of mice with CLP-induced sepsis. This result is in agreement with those of previous studies 61-65. As the percentage of T cell lineage population was greater in the spleen than in blood, further experiments were carried out in the spleen. Animals were euthanized 30 h after CLP induction. CD3^+^CD4^+^ T cell and CD3^+^CD8^+^ T cell counts were reduced in response to CLP induction, and CD3^-^CD19^-^NK1.1^+^ T cell counts were increased (Figure 8B). However, the population of T lineage cells remained unchanged upon treatment with ONB/MPA, and treatment with CLP, CLP+MPA, ONB, and CLP+ONB/MPA did not show any significant variation. These results indicate that the effect of ONB/MPA treatment on sepsis in our CLP-induced sepsis model does not involve immune cells belonging to the T cell lineage.

In this study, we evaluated the role of ONB/MPA with respect to controlled immunosuppression by studying human-derived PBMCs (*in vitro*) and LPS- and CLP-induced sepsis models (*in vivo*). Combining oxygen and an immunosuppressive drug is a novel idea for downregulating HIF-1ɑ and reversing hypoxia for controlling inflammation, and phospholipid-shelled ONBs were shown to be a viable carrier for MPA. Based on the results, we believe that ONB/MPA also has the potential to successfully control immunosuppression during implantation and grafting.

## Conclusion

In this study, the immunosuppressive application of ONBs was investigated by delivering the immunosuppressive drug MPA, while being encapsulated in ONBs. During normal and hypoxic conditions—established *in vitro*—immunosuppression due to ONB/MPA was observed in PMBCs. When ONB/MPA treatment was employed in murine models of LPS- and CLP-induced sepsis, we observed significant downregulation of growth factors and cytokines (i.e., IL-2, IL-6, and TNF-α). This controlled inflammation and significantly increased the survival rate of animals. Additionally, the biodistribution of fluorescent ONB/Cy5.5 indicated that ONBs are present in body 24 h after intravenous injection and 72 h after intraperitoneal injection, thus enhancing the bioavailability of the drug. ONBs were shown to be a non-toxic and effective drug delivery vehicle for immunosuppressive and hydrophobic MPA. This method can be potentially used for implantation and for the treatment of autoimmune diseases, wherein immunosuppression is desired.

## Methods

### Materials

1,2-Distearoyl-*sn*-glycerol-3-phosphocholine (DSPC), 1,2-distearoyl-*sn*-glycero-3-phosphoethanolamine-N-[biotinyl(polyethylene glycol)-2000] (ammonium salt) (DSPE-PEG-2000-Biotin), and 1,2-distearoyl-*sn*-glycero-3-phosphoethanolamine-N-[amino(polyethylene glycol)-2000] (ammonium salt) (DSPE-PEG-2000-Amine) were purchased from Avanti Polar Lipids (Alabaster, AL). IL-2, IL-6, and TNF-α ELISA kits were purchased from BioLegend (San Diego, CA). Cryopreserved human PBMCs were purchased from Cellular Technology Ltd (Cleveland, OH). FITC-avidin was purchased from Thermo Fisher Scientific (Waltham, MA). Dulbecco's phosphate-buffered saline (DPBS), chloroform, LPS, and MPA were purchased from Sigma Aldrich (St. Louis, MO). The anti-HIF-1ɑ antibody was purchased from Santa Cruz Biotechnology, Inc. (Dallas, TX).

### Synthesis of MPA-oxygen nanobubbles

The detailed composition of oxygen nanobubbles, and information regarding synthesis techniques and characterization methods have been described previously 13,49. To synthesize MPA-loaded ONBs (ONB/MPA), DSPC, DSPE-PEG-2000-Amine, and DSPE-PEG-2000-Biotin were dissolved in chloroform at a molar ratio of 85:8:7, and 10 mg MPA was added to the solution. Chloroform was then evaporated by placing the solution in a dry air oven. This process resulted in the formation of a thin dried layer, which was rehydrated by adding 10 mL DPBS and sonicating at temperatures above 50 °C using a bath-tub sonicator. This produced a liposomal suspension containing dissolved MPA (MPA concentration was 1 mg/mL). ONBs with 2 mg/mL MPA were also prepared for different treatment groups. The suspension was again sonicated using a tip sonicator at 190 W in pulsed mode for 5 min in the presence of oxygen to synthesize ONB/MPA. To remove the unconjugated drug, dialysis was performed against deionized water for three days using 1.4 kDa dialysis tubing. The amount of MPA in ONB/MPA was measured by observing fluorescence intensity according to the standard curve of MPA (360/420 nm). After the initial experiments, wherein DSPE-PEG-Biotin was found to exert inflammatory effects, the remaining experiments were performed by synthesizing ONBs using DSPC and DSPE-PEG-Amine at a molar ratio of 85:15.

### Characterization of ONB/MPA

NTA (Nanosight NS300, Malvern, USA) was used to measure the quantity of ONB/MPA. Samples were diluted 1:1000 in PBS. Optical and fluorescence microscopy, SEM (Carl Zeiss, Germany), and TEM (LIBRA 120, Carl Zeiss, Germany) were used to characterize ONB/MPA. SEM samples were made by pouring ONB on a glass slide and drying it using liquid nitrogen followed by Pt coating. Uranyl acetate was used to negatively stain ONB for TEM images at 80 kV.

### Cytotoxicity assay

Cytotoxicity assays were performed using the breast cancer cell line, MDA-MB-231 and PBMCs. Cells were seeded at 5 × 10^4^ cells/well in a 96-well plate and grown to 80% confluence, after which they were treated with (0, 1, 5, 10, and 50 µL/mL) ONBs and lipid solution over 24 to 48 h. Cell viability was measured using the cell counting kit-8 (CCK-8) assay. PBMCs were thawed and then cultured for 16 h prior to the experiments. PBMCs (1 × 10^5^ cells/well) were seeded in a 96-well plate containing Roswell Park Memorial Institute (RPMI) 1640 supplemented with 10% fetal bovine serum and 1% penicillin and streptomycin. They were then incubated in an atmosphere of 5% CO_2_. The numbers of live and dead cells were counted via CCK-8 assay at 450 nm.

### Drug release profile

The MPA content of ONBs was calculated from the MPA standard curve, plotted using MPA fluorescence curves at 360/430 nm. Peaks at 430 nm were recorded for the MPA standard curve. Drug release was calculated using the dialysis method at 37°C. ONB/MPA (1 mL) was injected into the dialysis tube, and it was placed in a 50 mL conical tube containing 10 mL DPBS. Data were recorded at time intervals of 0, 1, 3, 6, 18, 48, 96, and 144 h. After each time interval, 1 mL DPBS was removed and replaced with fresh DPBS, and fluorescence was measured to quantify the MPA released.

### Cytokine profiling

The release of human IL-2, L-6, and TNF-α from PBMCs was evaluated using ELISA (all reagents were obtained from R&D Systems). PHA, PMA (5 µg/mL of each), and LPS (1 µg/mL) were used as stimulants in different experiments. PBMCs (5 × 10^5^) /mL were cultured for 48 h, and after treatment, the cytokine release was measured after 48 h.

### Hypoxia

PBMCs were thawed and cultured for 16 h under standard conditions of 5% CO_2_ at 37 °C. Hypoxic conditions were created in a hypoxia chamber (20 × 30 × 30 cm) with hypoxic gas (5% oxygen, 5% CO_2_, and 90% N_2_). Next, 1 × 10^5^/mL PBMCs were seeded in a 96-well plate. The chamber was treated with mixed gas for 5 min to generate hypoxic conditions. Subsequently, the chamber was incubated for 24 h in standard incubator at 37 °C. The cells were then treated under different conditions and with varying concentrations of MPA, liposome, ONB/MPA, and ONBs. Finally, the supernatant was collected and evaluated for cytokine release after 48 h of incubation at 5% CO_2_, and 37 °C.

### *In vivo* model

All animal-related procedures were reviewed and approved by the Institutional Animal Care and Use Committee of the Hanyang University (protocol 2019-0081). LPS and CLP-induced sepsis models were used for the *in vivo* mouse model. MPA (1 mg/mL) was used for both ONB/MPA and MPA (control group). MPA was first dissolved in dimethyl sulfoxide (DMSO) and then in DPBS to a final concentration of 1 mg/mL. ONB/MPA was synthesized in the same manner as described above, although the dialysis step was skipped to maintain the MPA concentration identical to that in the ONB/MPA formulation (1 mg/mL). LPS injection (30 mg/kg) or the CLP procedure was performed 6 h prior to treatment, and treatment was performed intraperitoneally at 6, 12, and 18 h (n=15 for each of the five groups). The survival rate of the mice was monitored 100 h after LPS and CLP injection. The inflammation scores for lung and liver tissue were determined, and histological analysis was also performed 19.

### In vivo imaging

ONB/Cy5.5 was prepared by adding streptavidin-conjugated Cy5.5 dye to ONBs. We added 50 µg of dye per 1 mL of ONBs, and the sample was centrifuged at 300 x *g* for 10 min. *In vivo* imaging was performed using the IVIS Spectrum-CT *in vivo* imaging system. First, ONB/Cy5.5 were injected into the tail vein of the mouse, and the fluorescence was examined in spleen, liver, lung, and kidney tissues after 10 min, 30 min, 1 h, 3 h, 6 h, 24 h, 48 h, and 72 h. Additionally, ONB/Cy5.5 were injected intraperitoneally at 6, 12, and 18 h intervals. Fluorescence was observed at 18, 24, 48, 72, 96, and 120 h.

### Immunohistopathology

IHC of the lung, liver, spleen, kidney, and large intestine tissues was carried out. We examined and assigned a HIS corresponding to the severity of inflammation. We used a validated scoring system 66,67 in which a board-certified pathologist independently scored each organ section without prior knowledge of the treatment groups. A histological score ranging from 0-15 was ascribed to each specimen.

### Adaptive immune cell analysis

We examined the percentage of CD3^+^CD4^+^ T cells, CD3^+^CD8^+^ T cells, and CD3^-^CD19^-^NK1.1^+^ T cells in the blood or spleen over 4 days. This was done using FACS analysis in the background of CLP-induced sepsis. The animals were euthanized after 30 h of CLP induction, and the percentage of CD3^+^CD4^+^ T cells, CD3^+^CD8^+^ T cells, and CD3^-^CD19^-^NK1.1^+^ T cells was evaluated.

### Statistical analysis

GraphPad Prism software was used for statistical analysis and graphical representations of data. We performed Students* t*-tests and one-way analysis of variance (ANOVA) followed by post-hoc analysis to evaluate significance. For survival analysis, data were analyzed using the product-limit method proposed by Kaplan and Meier and the log-rank (Mantel-Cox) test (Prism, version 5.0, GraphPad Software). Non-significant values have been represented as ns, while *, **, *** indicate p-values < 0.5, 0.01, and 0.001, respectively.

## Supplementary Material

Supplementary figures.

## Abbreviations

MPA: Mycophenolic acid
ONB: Oxygen nanobubble
PBMCs: Peripheral blood mononuclear cells
HIF1-α: Hypoxia inducible factor-alpha
TNF-α: Tumor necrosis factor alpha
IL: Interleukin, TGF-β, Transforming growth factor beta, VEGF, Vascular endothelial growth factor, ELISA, Enzyme-linked immunosorbent assay
PCR: Polymerase chain reaction, DMSO, Dimethyl sulfoxide
CLP: Cecal ligation procedure
LPS: Lipopolysaccharide, PMA, Phorbol-12-myristate-13-acetate
PHA: Phytohemagglutinin
