# Supplementary material for: Effective delivery of mycophenolic acid by oxygen nanobubbles for modulating immunosuppression
Source: Theranostics. 2020 Mar 4;10(9):3892–904. doi: 10.7150/thno.41850 (PMC7086369; doi:10.7150/thno.41850)
Supplement: Supplementary file 1 — Supplementary figures. [file thnov10p3892s1.pdf]

Supporting Information

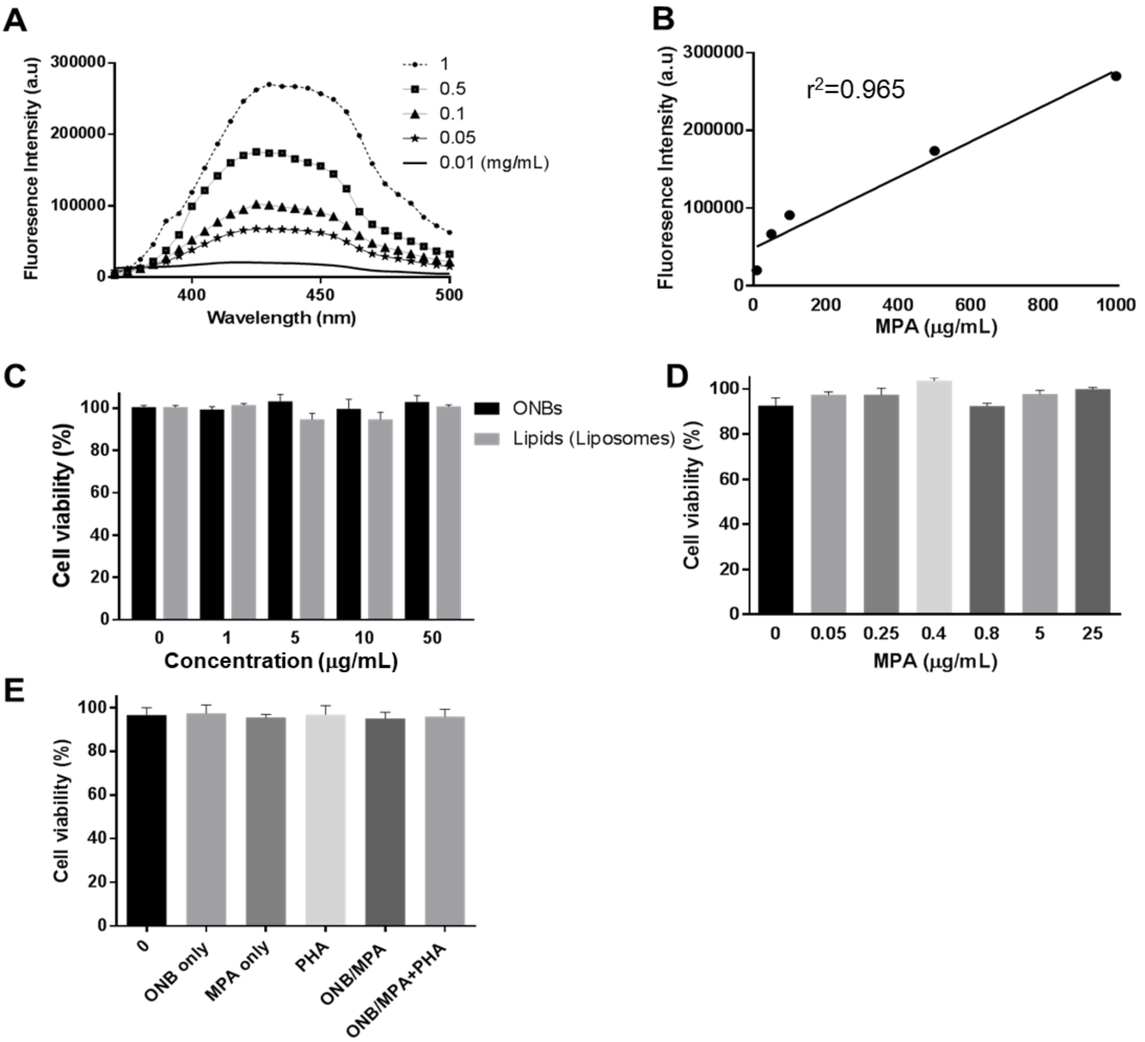

Figure S1. (A) Measurement of mycophenolic acid (MPA) fluorescence intensity based on various concentrations. (B**)** Measurement of standard curve of MPA/oxygen nanobubbles (ONBs), obtained from A (wavelength at 430 nm). (C) Viability of peripheral blood mononuclear cells (PBMCs) after treatment with ONBs and lipids (liposomes) (Trypan blue assay). (D) Viability of PBMCs after treatment with various concentrations of MPA. (E) Viability of PBMCs after treatment with a combination of ONBs and MPA.

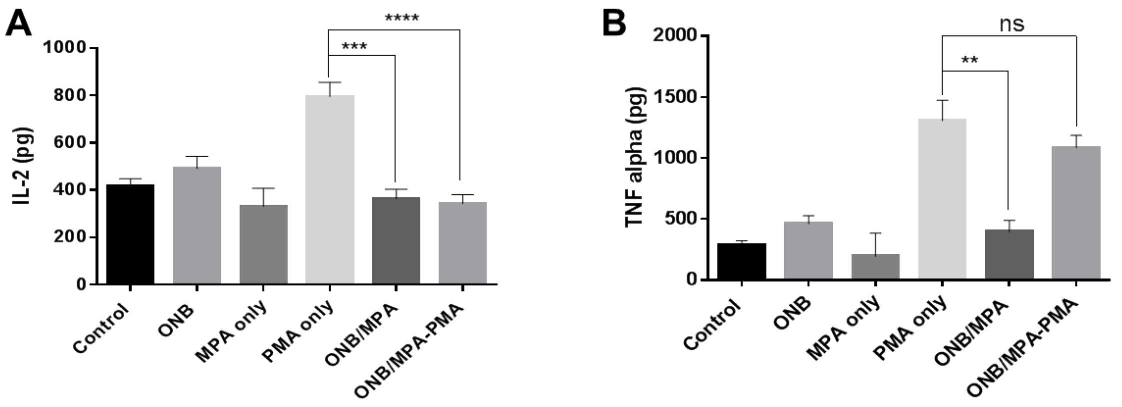

Figure S2. Cytokine profiling of ONBs without DSPE-PEG-Biotin. (A) IL-2 profile of ONBs, mycophenolic acid (MPA), phorbol 12-myristate 13-acetate (PMA), MPA/ONBs, and ONB/MPA/PMA-treated peripheral blood mononuclear cells (PBMCs). (B) TNF-α profile of PBMCs treated with ONBs, MPA, PMA, MPA/ONBs, and ONB/MPA/PMA (ONBs = 10%, MPA = 500 ng/mL).

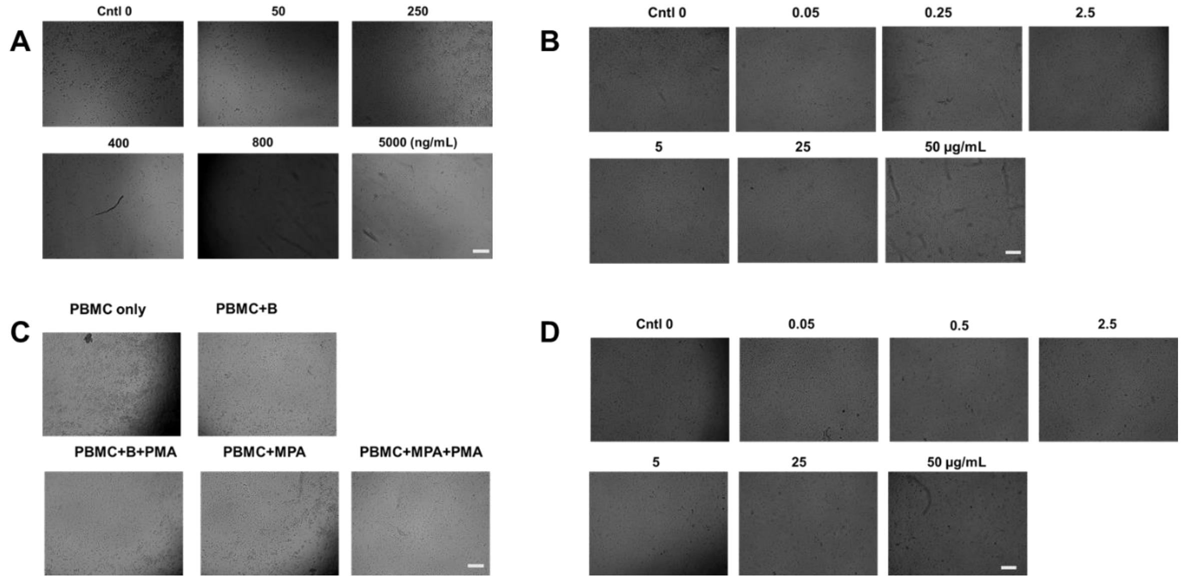

Figure S3. (A) MPA-treated PBMCs. (B) ONB-treated PBMCs **(**PBMCs = 5 x 10^5 cells/well, MPA = 50–5000 ng/mL). **(**C) Drug combination-treated PBMCs. PBMCs = 4 x 10^5 cells/well, ONBs = 20 µL/mL, PMA = 5 µg/mL, MPA = 500 ng/mL, ONB/MPA = 20 µL. (D) Lipid-treated PBMCs. PBMCs = 5 x 10^5 cells/well, ONBs = 0–50 µg/mL. All images were captured after adding MPA and incubating for 48 h. Scale bars = 100 µm.

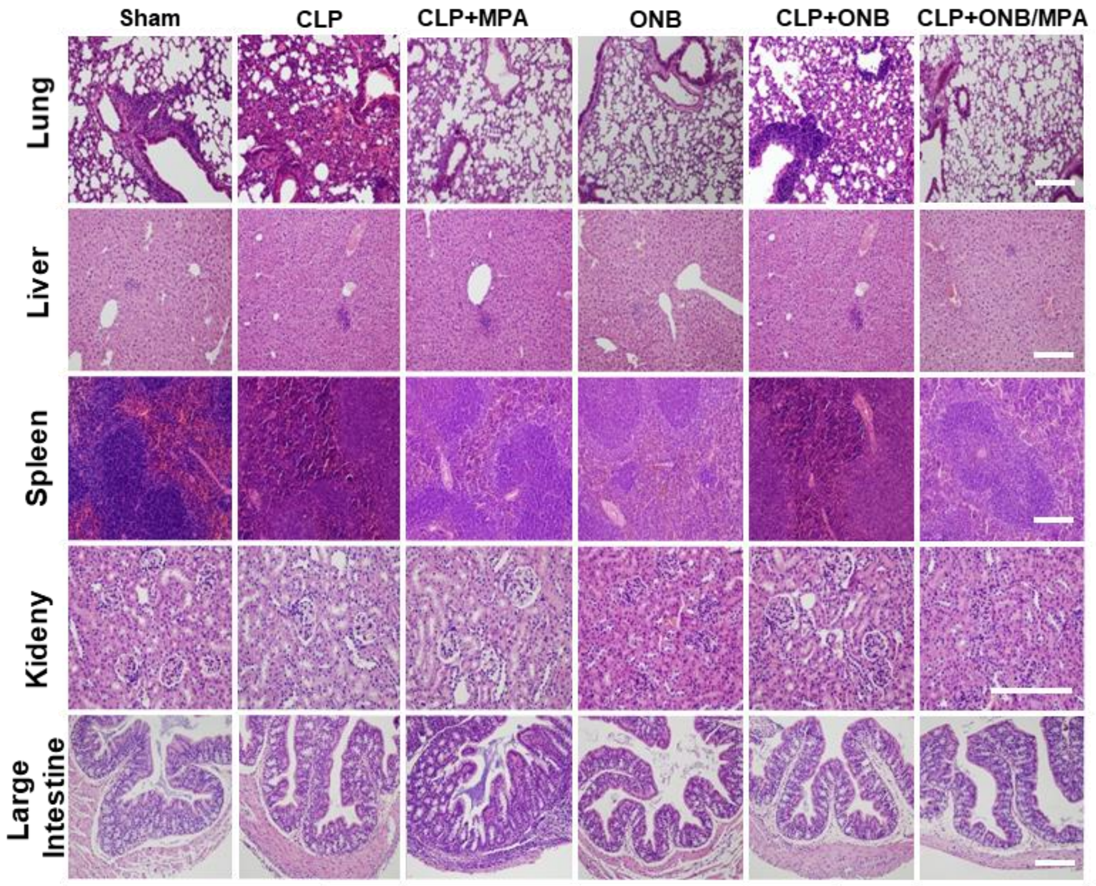

Figure S4. **Histological analysis of *in vivo* models.** ONB/MPA protects mice from cecum ligation and puncture (CLP)-induced sepsis. Representative hematoxylin and eosin (H&E) staining of lung, liver, spleen, kidney, and large intestine tissues (n=10 mice per group). Histopathological scores were obtained from H&E-stained tissue, as described in the Methods. Scores were determined 30 h after treating CLP mice with MPA, ONBs, or ONB/MPA. Please refer to Figures 4D and 7. Scale bars = 200 μm.
